# Imputation of canine genotype array data using 365 whole-genome sequences improves power of genome-wide association studies

**DOI:** 10.1371/journal.pgen.1008003

**Published:** 2019-09-16

**Authors:** Jessica J. Hayward, Michelle E. White, Michael Boyle, Laura M. Shannon, Margret L. Casal, Marta G. Castelhano, Sharon A. Center, Vicki N. Meyers-Wallen, Kenneth W. Simpson, Nathan B. Sutter, Rory J. Todhunter, Adam R. Boyko

**Affiliations:** 1 Department of Biomedical Sciences, College of Veterinary Medicine, Cornell University, Ithaca, New York, United States of America; 2 Cornell Center for Astrophysics and Planetary Science, Cornell University, Ithaca, New York, United States of America; 3 Department of Horticultural Science, University of Minnesota, St Paul, Minnesota, United States of America; 4 School of Veterinary Medicine, University of Pennsylvania, Philadelphia, Pennsylvania, United States of America; 5 Department of Clinical Sciences, College of Veterinary Medicine, Cornell University, Ithaca, New York, United States of America; 6 Baker Institute for Animal Health, College of Veterinary Medicine, Cornell University, Ithaca, New York, United States of America; 7 Biology Department, La Sierra University, Riverside, California, United States of America; Stanford University School of Medicine, UNITED STATES

## Abstract

Genomic resources for the domestic dog have improved with the widespread adoption of a 173k SNP array platform and updated reference genome. SNP arrays of this density are sufficient for detecting genetic associations within breeds but are underpowered for finding associations across multiple breeds or in mixed-breed dogs, where linkage disequilibrium rapidly decays between markers, even though such studies would hold particular promise for mapping complex diseases and traits. Here we introduce an imputation reference panel, consisting of 365 diverse, whole-genome sequenced dogs and wolves, which increases the number of markers that can be queried in genome-wide association studies approximately 130-fold. Using previously genotyped dogs, we show the utility of this reference panel in identifying potentially novel associations, including a locus on CFA20 significantly associated with cranial cruciate ligament disease, and fine-mapping for canine body size and blood phenotypes, even when causal loci are not in strong linkage disequilibrium with any single array marker. This reference panel resource will improve future genome-wide association studies for canine complex diseases and other phenotypes.

## Author summary

Complex traits are controlled by more than one gene and as such are difficult to map. For complex trait mapping in the domestic dog, researchers use the current array of 173,000 variants, with only minimal success. Here, we use a method called imputation to increase the number of variants–from 173,000 to 24 million–that can be queried in canine association studies. We use sequence data from the whole genomes of 365 dogs and wolves to accurately predict variants, in a separate cohort of dogs, that are not present on the array. Using dog body size, blood phenotypes, and a common orthopedic disease that involves rupture of the cranial cruciate ligament, we show that the increase in variants results in an increase in mapping power, through the identification of new associations and the narrowing of regions of interest. This imputation panel is particularly important because of its usefulness in improving complex trait mapping in the dog, which has significant implications for discovery of variants in humans with similar diseases.

## Introduction

The modern domestic dog (*Canis lupus familiaris*) consists of over 500 breeds selected for diverse roles and subject to wildly different disease prevalences [1]. A high quality reference genome [2–4] and affordable SNP genotyping arrays [5] have helped make the dog a powerful animal model for studying the genetics of complex traits and diseases. Of 719 genetic traits and disorders in the dog, 420 are potential models of human disease (https://omi.org/home/). With an average spacing of 1 SNP every 13kb, the CanineHD array (Illumina, San Diego, CA) has been successfully implemented in many genome-wide association studies (GWAS), especially within single breeds where linkage disequilibrium (LD) often extends beyond 1Mb [for example, see [6,7]. However, the results of many complex disease mapping studies in dogs have been underwhelming, with only one or a few significant loci identified [for example, see 8–10]. 57% of the 719 genetic traits and disorders in dogs are complex but the likely causal variant is known for only 27% of these (https://omia.org/home/).

Recently, we used simulations to show that an increase in SNP density to 1 SNP every 2kb would improve power for canine complex trait GWAS [8]. An increase in density can be achieved by the following: adding more SNPs to the CanineHD array, using whole genome sequencing (WGS), or using imputation to predict genotypes through the use of a reference panel created from WGS data. Of these, imputation is the most cost-effective option and has been used successfully in human and cattle GWAS, especially with the recent WGS efforts in these species [11,12]. Imputation has also been used in a canine within-breed GWAS of primary hypoadrenocorticism in the Standard poodle, resulting in an approximately 20-fold increase in SNP number, although this did not lead to the identification of a significant association [13].

GWAS of canine morphological traits has been very successful, due to large effect sizes and long regions of LD as a result of recent selection in purebred dogs [14]. Seventeen quantitative trait loci (QTLs) associated with body weight, as a proxy for body size, have been identified [5,8,15–23], as well as associations for other morphological phenotypes such as ear flop [5,16,17] and fur type [8,24,25]. Despite the success of morphological trait mapping, we suggest that imputation can improve the power of GWAS, especially for reducing large intervals for use in fine-mapping.

In addition to morphological traits, we aim to improve on complex canine disease associations and blood phenotype associations recently conducted using the CanineHD array. For diseases, we focus on the orthopedic disease of cranial cruciate ligament disease (CCLD), a complex genetic disease that involves the partial to complete rupture of the cranial cruciate ligament and is a well-established model for anterior cruciate ligament rupture in humans [26]. Several recent studies have identified significant associations [26–30], mostly within high-risk breeds and none of these results overlap. For blood phenotypes, we recently performed an across-breed GWAS of 353 dogs on 39 blood phenotypes (complete blood count and clinical chemistry panel), resulting in 9 phenotypes that yielded significant associations [31].

We posit that improving the density of variants by using an imputation panel will greatly improve the power to identify causal loci for canine complex traits, due to increased LD. We use 365 canine whole genome sequences to create a reference panel of 24 million variants and impute these variants in 6,112 dogs previously genotyped on a semi-custom 185k CanineHD array. We show that using an imputation panel increases our power to detect variants affecting complex canine traits–including a common orthopedic disease, CCLD–by identifying potentially novel loci, and by refining intervals for previously-identified QTLs for use in fine-mapping. To our knowledge, this is the first study to use an imputation panel based on WGS for across-breed canine mapping studies.

## Results

### Evaluation of imputation accuracy

**Fig 1:**
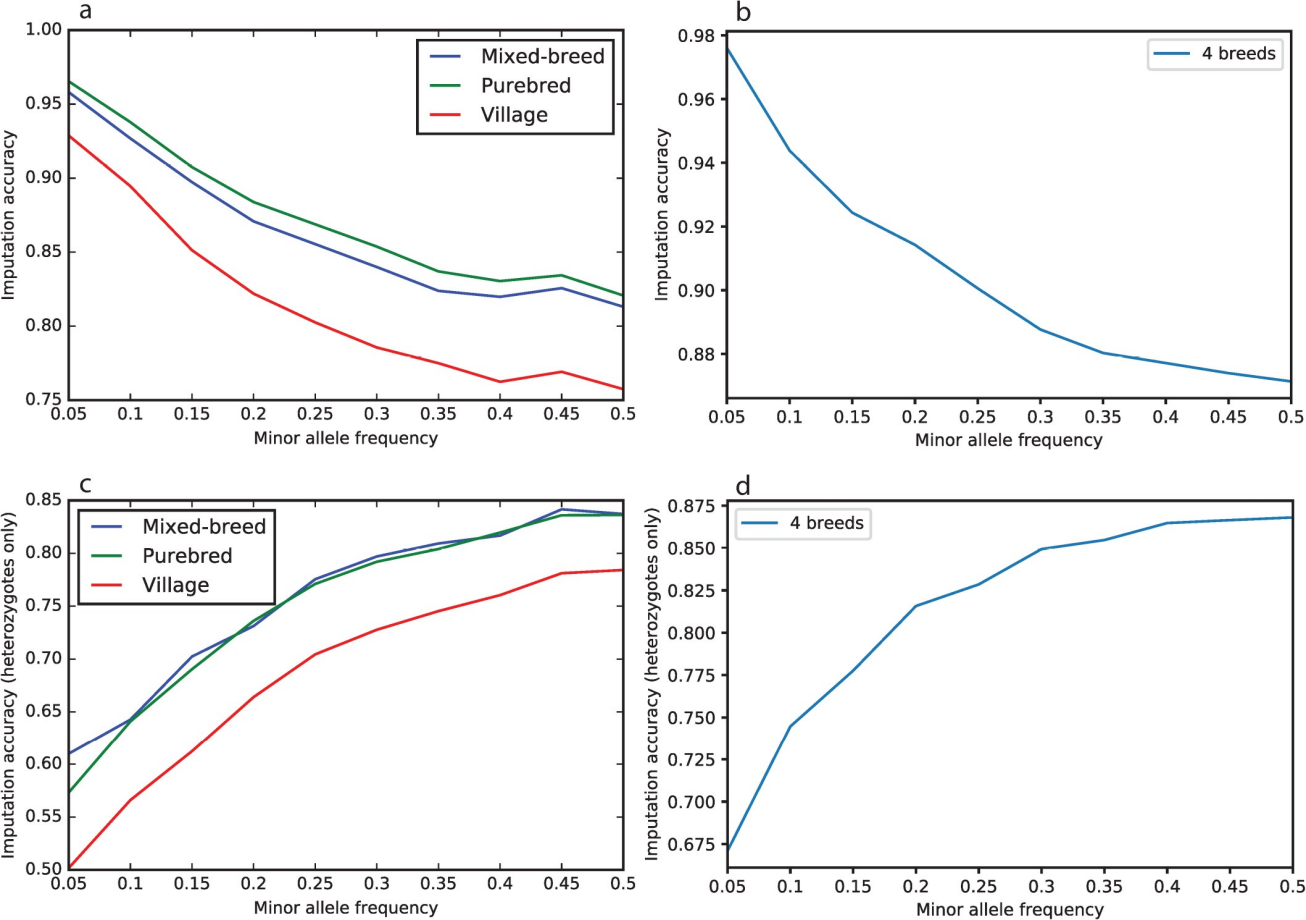
Imputation accuracy for 33,144 variants of different allele frequency. a) All sites, c) heterozygous sites only in mixed-breed dogs (blue), purebred dogs (green), and village dogs (red), and in the four most represented breeds in the WGS reference panel for b) all sites, d) heterozygous sites only.

We used IMPUTE2 to impute the WGS reference panel across the 6,112 genotyped dogs resulting in 24 million variants. By comparing 33,144 imputed variants to directly genotyped sites on a second custom array, we were able to calculate the accuracy for our imputation panel, which was 88.4% overall. Across all sites, purebred dogs had the highest accuracy (89.7%, n = 276), followed by mixed-breed dogs (88.6%, n = 13), and then village dogs (84.2%, n = 86). This result is expected given that 210 of our 365 WGS panel were purebred dogs, and also due to the long-range haplotypes found in purebreds that make calling imputed variants easier. For all three dog types (purebred, mixed-breed, and village), imputation accuracy increased with decreasing minor allele frequency (MAF) (Fig 1A), which is an expected result because as MAF decreases, the occurrence of the major allele is the correct call more often. Looking at true heterozygous sites only (Fig 1C), imputation accuracy was lower across most MAFs compared to all sites (Fig 1A). Imputation accuracy increased as MAF increased for heterozygous sites, as there are more heterozygous calls for SNPs with higher MAF.

Looking at the imputation accuracy in the four breeds with the highest number of dogs in the WGS panel (Yorkshire terriers, Maltese, German shepherd dog, and Labrador retriever), the overall imputation accuracy was 92.7% (n = 32), a 3% increase from the larger panel of 276 purebred dogs described above. The same trends of increasing accuracy with decreasing MAF and vice versa for heterozygous sites are observed for the four breeds analysis but at higher imputation accuracies (Fig 1B–1D).

In general, the larger chromosomes and chromosome X had higher imputation accuracies than the smaller chromosomes, such as 35, 36, 37, and 38, due to lower recombination rates (S1 Table). For all purebred, mixed-breed, and village dogs, the average imputation accuracy per chromosome was 89.1% (range of 84.3–93.5%), 88.0% (range of 83.0–93.0%), and 83.7% (range of 78.5–92.4%), respectively.

### Body size associations

We performed two separate GWAS, firstly using the semi-custom CanineHD array data of 185k markers, and secondly using our imputed panel of 24 million variants. The phenotypes used in these GWAS were male breed-average weight, male breed-average height, and individual sex-corrected weight. We were then able to compare the results from the array GWAS and the imputed GWAS using the exact same phenotypes (see Table 1 for male breed-average weight results). In the imputed GWAS for male breed-average weight, we identified 20 significant QTLs, 5 of which were potentially novel, and of the 17 previously identified body size loci only two (CFA3:62 and CFA12:33) were not significant (S2 Table). For the imputed analysis of male breed-average height, we identified 16 significant QTLs, 3 of which were potentially novel, and we saw 13 of the known 17 body size loci (S2 Table). Finally, for the imputed GWAS of individual sex-corrected weight, we found 12 significant QTLs, 2 of which were potentially novel, and we found 10 of the known 17 body size loci (S2 Table). Note that the CFA12:33 QTL was not significant in the male breed-average weight imputed GWAS (*P* = 1.3×10^−8^) but was significant in the male breed-average height imputed GWAS (*P* = 2.8×10^−22^).

**Table 1:** SNPs associated with male breed-average weight using the array data and imputed data.

Chr	Putative causal variant	Array Position	Array MAF	Array Effect size	Imputation Position	Imputation MAF	Imputation Effect size	LD (r^2^)
1	N/A	**55983871**	0.129	-0.071	**55922563**	0.127	-0.077	0.874
3	41849479	**41758863**	0.311	-0.031	**41780841**	0.111	-0.074	0.246
3	N/A	**62042184**	0.100	0.062	61887587	0.093	0.065	0.849
3	N/A	**91103945**	0.227	0.067	**91110878**	0.283	0.094	0.433
4	39182836	**39112085**	0.334	-0.052	**39182836**	0.262	-0.060	0.396
4	67040898 67040939	**67026055**	0.404	-0.033	**67040898**	0.363	-0.074	0.382
5	N/A	**31895829**	0.461	-0.029	**31689208**	0.248	-0.045	0.265
7	N/A	**30243851**	0.271	-0.044	**30183217**	0.176	-0.067	0.518
7	N/A	**41392649**	0.356	-0.041	**41351722**	0.349	-0.044	0.903
7	43794129	**43719549**	0.382	-0.067	**43724293**	0.356	-0.076	0.894
9	N/A	N/A			**12034947**	0.054	-0.094	N/A
10	8348804	**8183593**	0.209	-0.135	**8379634**	0.249	-0.191	0.557
11	N/A	**26929946**	0.243	-0.045	**26929946**	0.243	-0.045	1.000
12	33710200	**33733595**	0.435	0.026	33712492	0.161	0.067	0.148
15	41221438 41220982	**41221438**	0.465	-0.115	**41216098**	0.464	-0.114	0.979
18	20443727	**20272961**	0.151	-0.083	**20379945**	0.161	-0.116	0.577
20	N/A	**21479863**	0.085	0.056	**21686712**	0.110	0.072	0.493
26	N/A	**7631562**	0.340	0.031	**7679257**	0.175	0.059	0.075
26	N/A	**13224865**	0.307	-0.042	**12838979**	0.232	-0.078	0.154
32	5231894	N/A			**5228269**	0.164	-0.077	N/A
34	N/A	**18559537**	0.237	-0.050	**18587956**	0.258	-0.051	0.849
39	102364864 102369488	**102212242**	0.345	0.073	**102209680**	0.342	0.075	0.983

Positions, minor allele frequencies (MAF), and effect sizes for SNPs associated with male breed-average weight using the array data and imputed data, and LD between these pairs of SNPs. Also shown are putative causal variants for loci that have previously been fine-mapped. 20 array QTLs and 20 imputed QTLs (shown in bold) were used in a prediction model.

**Fig 2:**
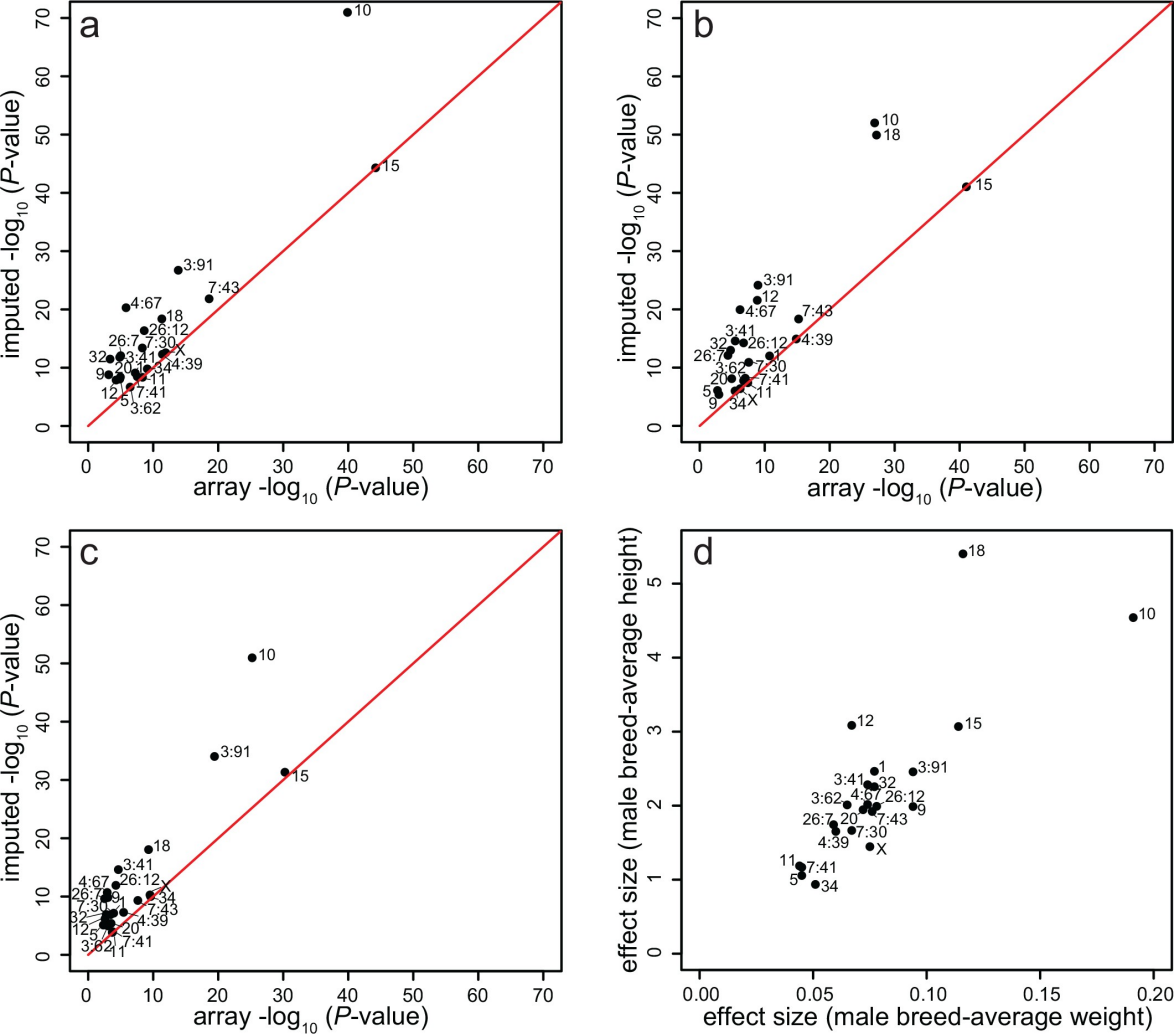
Scatter plots showing *P*-values and effect sizes for array vs imputed GWAS body size results. *P*-values for array GWAS (x axis) and imputed GWAS (y axis) results for the 22 body size QTLs (as shown in Table 1) a) male breed-average weight, b) male breed-average height, c) individual sex-corrected weight, and d) absolute values of the effect sizes of the most-associated SNP for male breed-average weight and male breed-average height from the imputed GWAS.

Using imputed data generally increased the significance of body size associations seen in the array data, especially *HMGA2* on CFA10 and fgf4 on CFA18 for height (Fig 2A, 2B and 2C; S2 Table). Most of the respective QTLs from the array GWAS and the imputed GWAS were in LD (r^2^ > 0.2) with the exception of the CFA12 and two CFA26 associations (Table 1). When imputed variants were not in high LD (r^2^ < 0.8) with array QTLs, the imputed variants generally had stronger effect sizes and lower minor allele frequencies (Table 1).

For most size-associated variants, the male breed-average weight and height effects were roughly isometric, with the exception of CFA18 and CFA12 QTLs, which had a greater effect on height than weight (Fig 2D). Of the seventeen QTLs that have been previously associated with body size [5,8,15–23,32], only one (CFA3:62) did not reach significance in any of the three imputed analyses (significance threshold of *P* = 1×10^−8^), with *P*-values of 1.1×10^−5^, 2.1×10^−7^ and 1.8×10^−8^ for individual sex-corrected weight, male breed-average weight and male breed-average height respectively (S2 Table). GWAS of male breed-average weight provided the most power on average, so we will focus on that phenotype for the rest of the body size analyses.

We used the significant body size QTLs from the male-breed average weight GWAS to predict the body weight of individuals, by randomly setting 20% of the body weights in the dataset to missing, then using a Bayesian sparse linear mixed model to predict the missing weights, and finally comparing the predicted weights to the actual weights. Using the 20 QTLs identified from the array GWAS (see bold in Table 1), we found a correlation coefficient (r) of 0.851 and this increased to 0.869 when we used the 20 QTLs from the imputed GWAS (see bold in Table 1).

### Known body size loci

Nine body size QTLs have previously been fine-mapped (*IGF1R*, *STC2*, *GHR*, *SMAD2*, *HMGA2*, *IGF1*, fgf4 retrogenes on CFA12 and CFA18, *IGSF1*) [18–21,32,33]. For each of these, the region in LD with the most significant respective marker in the male breed-average weight imputed GWAS contained the known or putative causal variant (S1 Fig). While the putative causal variants weren’t always the highest associated variant at a locus, they generally had *P*-values within two orders of magnitude of the most associated marker (S1 Fig), confirming that the imputation panel performs well for the weight GWAS.

Unsurprisingly, many of the putative causal variants are not markers on the CanineHD array, including *IGF1R* (3:41,849,479) [21], *STC2* (4:39,182,836), *GHR* (4:67,040,898 and 4:67,040,939), and *HMGA2* (10:8,348,804) [18]. With the array data, the *IGF1R* QTL (*P* = 1.4×10^−5^ for male breed-average weight GWAS) did not reach significance, but with the imputed data we saw a significant association signal (*P* = 1.6×10^−12^ for male breed-average weight GWAS), and the causal variant was the 6^th^ most associated SNP (r^2^ = 0.73 between causal and associated SNP) (S1A Fig). The *STC2* and *GHR* (4:67,040,898) putative causal variants were the most significant variants at those loci in the imputed GWAS (S1B and S1C Fig). Note that there are two putative causal variants for *GHR* [18], both in exon 5, but only one passed our 5% MAF filter. Similarly, the *HMGA2* causal variant was in high LD (r^2^ = 0.91) with the most significant marker at this locus in the imputed GWAS (S1E Fig).

For *IGF1*, SNP5 (BICF2P971192, 15:41,221,438), which is in LD with the SINE element [19], was the most significant association in the array GWAS. In the imputed GWAS, SNP5 was the 2^nd^ most associated SNP and the SNP that tags the SINE element (15:41,220,982) was the 4^th^ most associated SNP, and these SNPs were nearly in complete LD with the most significant marker in the GWAS (r^2^ = 0.98 and 0.97 respectively) (S1G Fig). The *IGSF1* missense mutation [33] was in high LD with the most significant association in the imputed GWAS (r^2^ = 0.97) (S1I Fig). Note that there is a second variant in *IGSF1* –an in-frame deletion–that has also been identified [33].

### Imputed GWAS potentially novel body size loci

Seventeen QTLs have been previously associated with body size in dogs [8]. Here, using the imputation data, we found a further five QTLs (CFA5:31, CFA7:41, CFA9:12, CFA26:7, CFA32:5) that passed our significance threshold in a male breed-average weight GWAS. As a conservative control, we performed a further male breed-average weight GWAS in which we included the four most-associated QTLs (CFA10:8, CFA15:41, CFA3:91, CFA7:43) as covariates. The results showed that the potentially novel loci at CFA5:31, CFA7:41 and CFA32:5 were no longer significant–and four other loci were also not significant in the covariate GWAS: CFA1:56, CFA11:26, CFA20:21, CFA34:18. Further analyses are required to determine if these are true or spurious associations, but since we cannot rule out that they are spurious, we conclude that we have identified only two potentially novel canine body size QTLs, at CFA9:12 and CFA26:7.

**Fig 3:**
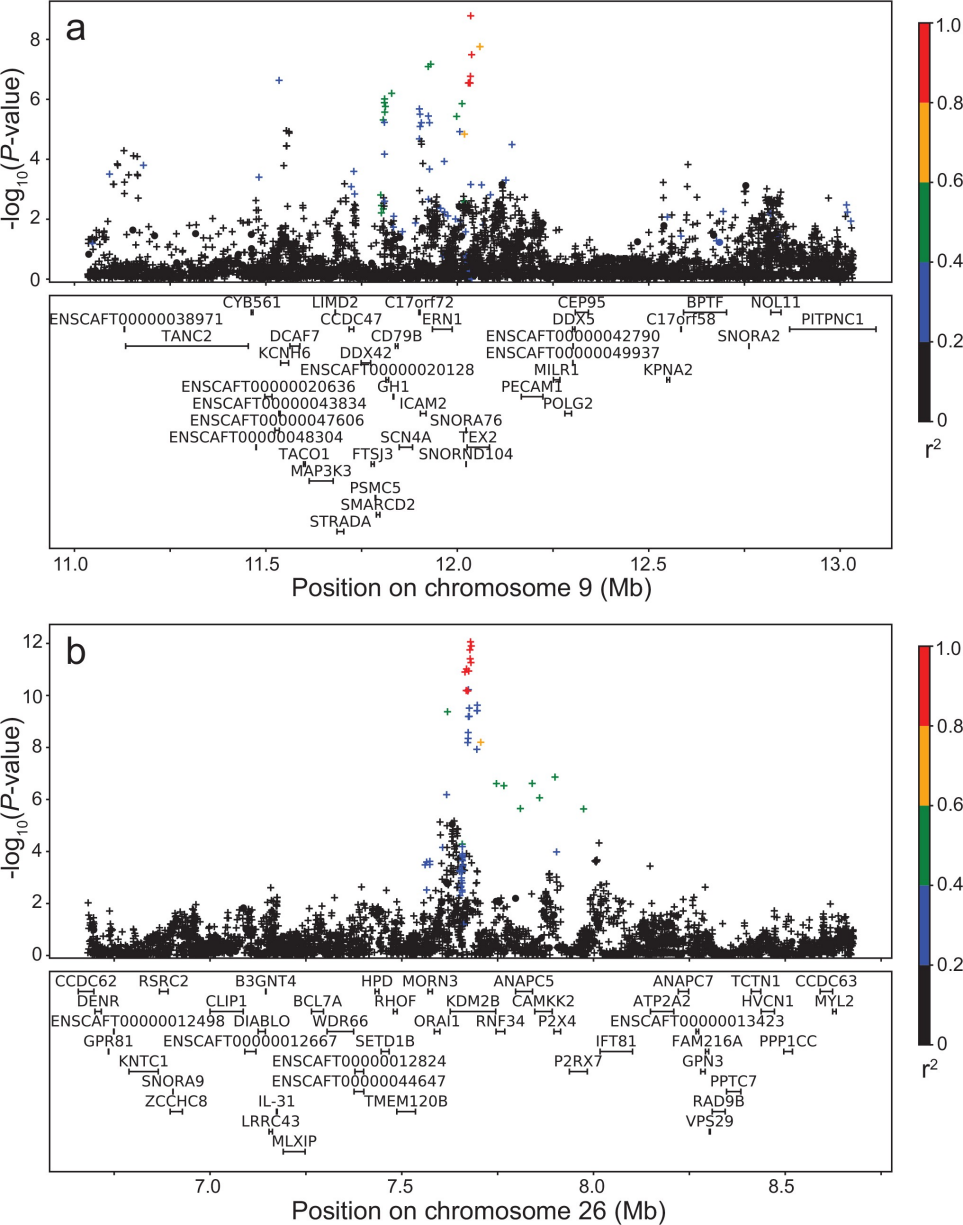
Potentially novel body size loci. Linkage disequilibrium plots of the region around male breed-average weight GWAS results a) CFA9:12, b) CFA26:7. Array genotypes are shown as o, imputed data are shown as +. Colors indicate amount of LD with the most significantly associated SNP, ranging from black (r^2^<0.2) to red (r^2^>0.8).

The most significant SNP at CFA9:12 is located about 200kb upstream of the gene *growth hormone 1* (*GH1)* (Fig 3A), which is expressed in the pituitary and has been associated with body size in humans and cattle [34–36]. The non-reference, derived indel that was the most highly associated in our imputed GWAS is found at high frequency in the small breeds Papillon, Yorkshire terrier, and Pomeranian, and also in New Guinea Singing Dogs (S3A Table). The genotype proportions show that the smallest dogs in our dataset have the highest proportion of the derived indel (S3B Table). Using our snpEff annotated variant files, we found two variants in *GH1*: a splice donor variant in intron 3 (CFA9:11,833,343, c.288+2_288+3insT), and an in-frame deletion in exon 5 (CFA9:11,832,437, c.573_578delGAAAGA, p.Lys191_Asp). Both of these variants were at <5% frequency in the WGS panel but all occurrences were in small-sized breeds (such as Yorkshire terrier and Maltese).

The second potentially novel body size QTL is at CFA26:7 (Fig 3B). Investigation of the surrounding region uncovered a couple of potential candidate genes. The first is *ANAPC5*, a member of the anaphase-promoting complex gene family that includes *ANAPC13*, which has been associated with height in humans [37]. The second candidate gene is the histone H3 demethylase *KDM2B* (*lysine-specific demethylase 2B)*, which has been associated with body mass index in humans in a CpG methylation study [38]. However, we did not identify any variants in *ANAPC5* or *KDM2B* in the snpEff-annotated files that are in LD with the associated imputation variant. The non-reference, derived allele was found at high frequency in the small breeds Shiba Inu, Papillon, and Chihuahua (S3A Table). The genotype proportions again show that the derived allele is most common in the smallest weight class in our dataset (S3B Table).

### Refinement of body size loci

With the imputation panel, we saw a refinement in several QTL regions–for example, the chromosome 3 association near the genes *LCORL* and *ANAPC13*, both of which have previously been associated with body size [5,16,36,37,39]. Using imputed data, this QTL had a more significant and defined association, compared to the CanineHD array data alone (S2A Fig). The QTL interval is about 65kb and 60kb upstream of the genes *LCORL* and *ANAPC13* respectively, suggesting the causal variant is likely regulatory. Another example is the recently identified body size QTL at CFA7:30 Mb, near the gene *TBX19* [8]. Here the imputed GWAS results showed a narrower QTL interval of greater significance when compared to the array GWAS (S2B Fig). This region overlaps *TBX19* but we did not observe any coding loci in our snpEff annotated variant files that are in LD with the most associated SNP.

### Allelic heterogeneity

**Fig 4:**
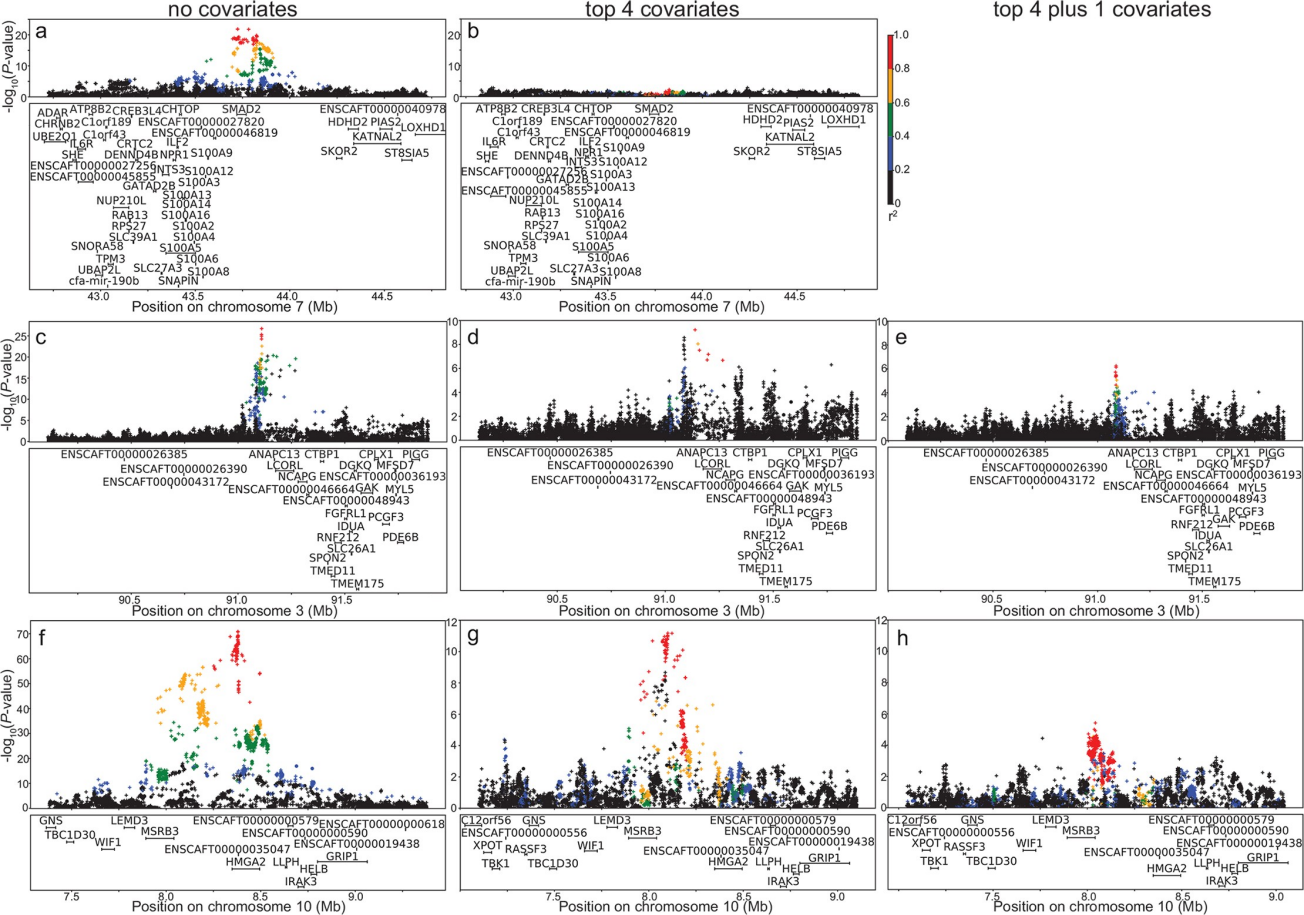
Stepwise-covariate LD plots for male breed-average weight imputed GWAS. a) CFA7 (*SMAD2*), showing that the association signal in the region disappears with the inclusion of the top 4 SNPs as covariates (as shown in b). Association signal in the region remains with the inclusion of the top 4 SNPs as covariates and then additional stepwise covariates in the region c)-e) CFA3 (*LCORL)*, f)-h) CFA10 (*HMGA2)*. Array genotypes are shown as o, imputed data are shown as +. Colors indicate amount of LD with the most significantly associated SNP, ranging from black (r^2^<0.2) to red (r^2^>0.8).

In order to reduce phenotypic noise, again we included the four most-associated QTLs (CFA10:8, CFA15:41, CFA3:91, CFA7:43) as covariates in the GWAS (hereafter referred to as “top 4 covariates”), and then implemented a region-specific stepwise approach, including further associated SNPs in the region as covariates, until no significant association signal remained. For male breed-average weight, when we regressed out the most significant association for a QTL, we expected the association signal to disappear, as seen with the *SMAD2* QTL (Fig 4A and 4B).

Our results showed two QTLs (CFA3:91, CFA10:8) that retain significant association signal after regressing out the most associated locus in the respective region (Fig 4C–4E and 4F–4H). For both CFA3 and CFA10, the data suggest there may be two independent significant associations in these regions. In the CFA3 region, the initial association signal peak looked regulatory while the residual signal is located in the genes *ANAPC13* and *LCORL*. The residual signal in the CFA10 region lies close to the ear flop association [5,16,17] (candidate gene *MSRB3*) but is not in LD with the imputed ear flop locus at CFA10: 8,097,650 (r^2^ = 0.147). The variant in this residual signal region may be regulatory, as the significance peak lies approximately 248kb upstream of *HMGA2*. We did not identify any coding variants in these two residual signal regions from the snpEff annotations. Two other QTLs (CFA4:67 and CFA15:41) showed evidence of residual signal but these did not reach significance (*P* = 2.9×10^−7^ and 2.9×10^−8^, respectively).

This residual signal suggested allelic heterogeneity in these regions but could also be due to imperfect tagging in the imputed dataset. As a follow-up analysis, for each of these two QTLs (CFA3:91 and CFA10:8), we took the most significant SNP from the top 4 covariates GWAS. We used that significant SNP as a covariate in a GWAS to see if we were able to recover the most significant SNP from the initial GWAS with no covariates. For both CFA3 and CFA10, we did recover the initial associated SNP, suggesting that these are real associations and not midway between two imperfectly tagged SNPs.

### Blood phenotypes

**Fig 5:**
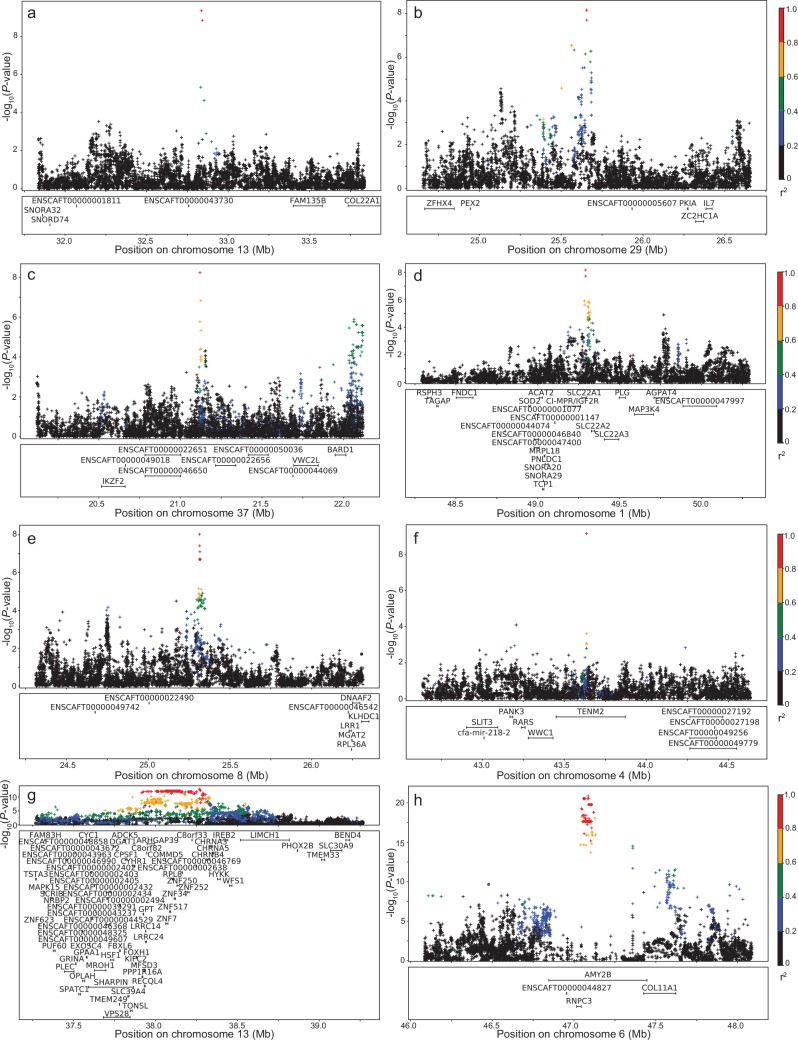
LD plots for significant blood phenotypes. a) albumin on CFA13, b) anion gap on CFA29, c) calcium on CFA37, d) sqrt glucose on CFA1, e) potassium on CFA8, f) white blood cells on CFA4, g) log ALT on CFA13, h) sqrt amylase on CFA6. Array genotypes are shown as o, imputed data are shown as +. Colors indicate amount of LD with the most significantly associated SNP, ranging from black (r^2^<0.2) to red (r^2^>0.8).

Using our imputed panel for GWAS on blood phenotypes revealed several potentially novel associations. For example, we saw significant associations with the phenotypes of albumin (Fig 5A) and calcium (Fig 5C) levels in peripheral blood (*P* = 4.5×10^−10^ and 5.9×10^−9^ respectively), neither of which were previously identified in the array GWAS [31] (S4 Table). We also identified a potentially novel association with blood glucose level and CFA1 (Fig 5D), located in the gene solute carrier family 22 member 1 (S*LC22A1)* and about 30kb downstream of the insulin-like growth factor 2 receptor gene *(CI-MPR/IGF2R)*. During gestation, *IGF2R* binds insulin-like growth factor 2 (IGF2), the presence of which stimulates the uptake of glucose [40]. The SNP was at highest frequency (>50%) in the Samoyed and American Eskimo dog breeds.

Of the eight significant associations (using a threshold of *P* = 1.0×10^−8^) we saw with the imputed data, only two were not novel–alanine aminotransferase (ALT) and amylase–although both increased in significance (Fig 5G and 5H; S4 Table). In addition to significant associations, we also saw six associations that nearly meet our significance threshold, that is, *P* < 1.8×10^−8^, including three that were not significant using the genotype data (S4 Table).

### Cranial cruciate ligament disease

**Fig 6:**
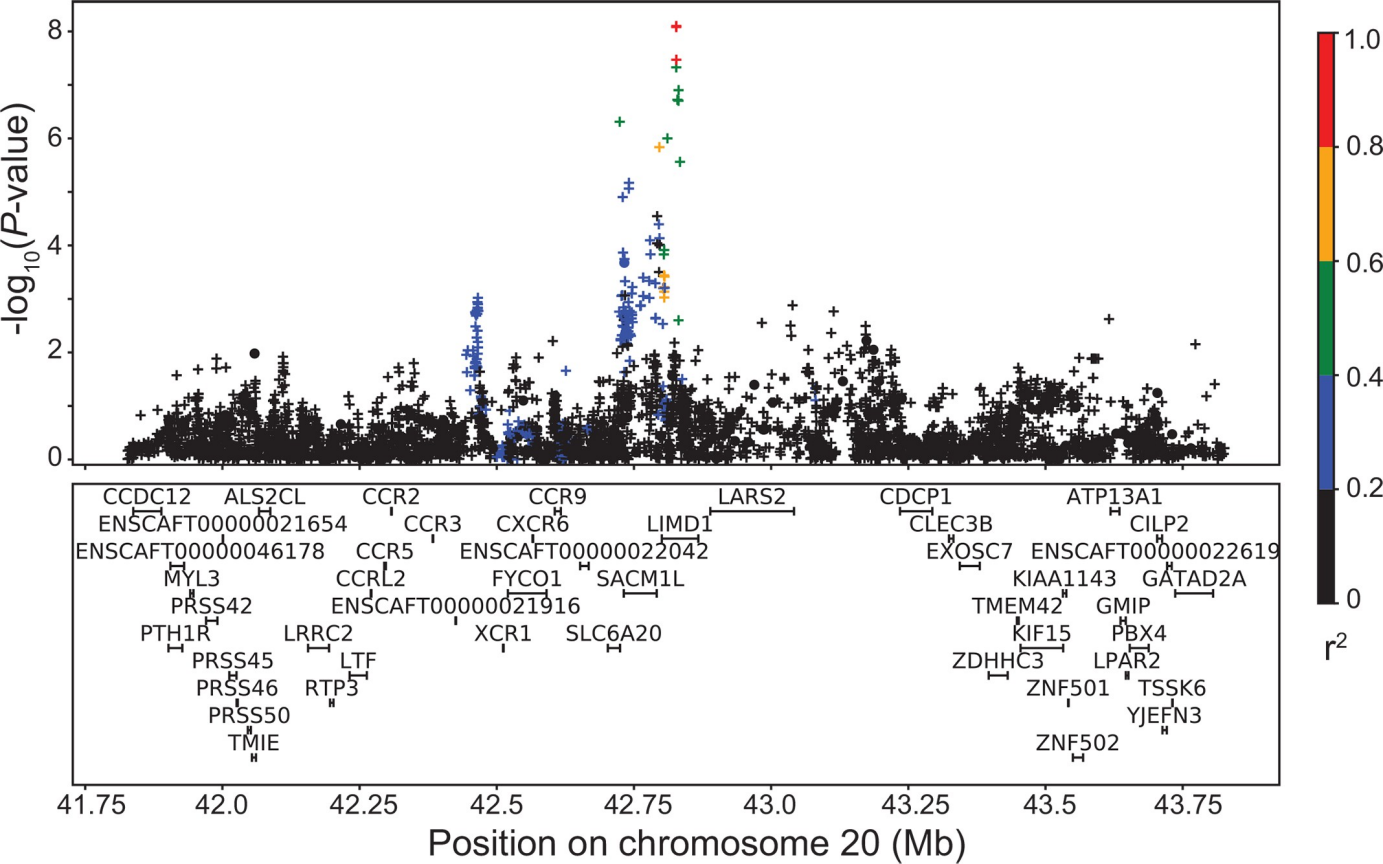
LD plot for significant CCLD association. Linkage disequilibrium plot of the region around the imputed CFA20 association with CCLD. Array genotypes are shown as o, imputed data are shown as +. Colors indicate amount of LD with the most significantly associated SNP, ranging from black (r^2^<0.2) to red (r^2^>0.8).

Using a quantitative GWAS design that separates the CCLD cases into partial rupture and complete rupture, we identified a significant association using the imputation data that is not identified using the array data (Fig 6). This association was located on CFA20: 42,827,199 (*P* = 7.9×10^−9^) in the gene *LIM Domain-Containing 1 (LIMD1)*, which is involved in regulating stress osteoclastogenesis, and osteoblast function and differentiation [41,42]. 10% of all dogs with a complete rupture (n = 84) have two copies of the minor allele, C, at this marker, while only 1% of all control dogs (n = 377) and 4% of all dogs with a partial rupture (n = 141) have two copies of the C allele (S5A Table). This locus has an effect on CCLD in some breeds (such as German shepherd dog and Labrador retriever) but not others (such as Golden retriever and Rottweiler) (S5B Table).

## Discussion

Imputation increases GWAS power by including additional sites that are not well-tagged by any single array marker, and has been successfully implemented in human studies, for example, low-density lipoprotein GWAS [43–45]. Here we present a canine imputation panel of 24 million variants–an approximate 130-fold increase in SNP number and SNP density from the semi-custom CanineHD array–for use in association studies. This panel has an overall accuracy rate of 88.4% when compared to genotype data from the same individuals (276 purebreds, 86 village dogs, and 13 mixed-breed dogs). It is important to note that our WGS reference panel includes only 76 breeds while our genotyping panel contains 185 breeds, so more than half the breeds we are imputing across are not represented in our reference panel. Furthermore, there is an average of only 2.7 dogs (SD 3.4, range 1–16) within a single breed in our reference panel. Both of these factors act to reduce the accuracy of our imputation. In the future, panels based on even larger numbers of sequenced individuals would yield even higher accuracy [for example, see 46], but our current panel based on hundreds of dogs is still a useful way to improve the power of canine mapping studies today.

Although the price of WGS is decreasing, it is still more cost-effective to use a panel of WGS individuals to create an imputation dataset based on genotyped samples than it is to directly WGS all samples [47]. Our imputation panel was created using over 350 canine WGSs representing 76 breeds. The inclusion of more breeds, especially diverse breeds (such as the Parson Russell Terrier) and rare breeds (such as the Pumi), will improve the accuracy of, and the number of rare variants in, future imputation panels. A recent canine imputation study has shown that imputation accuracy is highest using a multi-breed reference panel (compared to a breed-specific panel), and when there is overlap in breeds between the target and reference panel [48]. Indeed, the overall imputation accuracy rate here increased to 92.7% when including only individuals of the four breeds that are most highly represented in our reference panel, highlighting the usefulness of this resource for within-breed studies. Furthermore, human studies have shown that imputation accuracy increases with the size of the reference panel [49,50].

With our imputation panel, we improved association mapping for previously studied phenotypes, such as body size. Previous mapping studies of canine body size and other morphological traits using CanineHD array data have identified many significant QTLs. This success is largely the result of selection for body size during the formation of dog breeds, leading to selective sweeps around large-effect loci that facilitated mapping efforts. Nevertheless, using the imputation panel, we were able to identify two additional potentially novel loci (at CFA9:12 and CFA26:7) that influence body size. Using imputation, we were also able to narrow intervals for previously known associated QTLs, and find evidence of possible allelic heterogeneity at two loci. Furthermore, imputation provides a more accurate analysis of the genetic architecture underlying canine body size and, in turn, allows a more accurate prediction for body size in dogs.

Imputation is especially helpful in across-breed and/or mixed-breed study designs, where LD breaks down very rapidly making it more difficult to identify associations. Increasing the number and density of queried variants (as done by imputation) increases the chance that a variant will be in LD with the causal variant, especially when compared to a within-breed study design. We used our imputation panel for across-breed GWAS of blood phenotypes and the orthopedic disease CCLD. For blood phenotypes, we show several potentially novel associations and the narrowing of associated intervals when compared to array data alone, and for CCLD, we identified a significant association (at CFA20:42) that was not identified with array genotype data. Previously identified loci that influence CCLD risk include five in Labrador retrievers (CFA1,4,6,23,24), three in Newfoundlands (CFA1,3,33), one in Rottweilers (CFA9), and another two from an across-breed GWAS design (CFA8,9) using the same phenotyped dogs as this study [26–30]. This CFA20 potentially novel association is especially important, given that CCLD in the domestic dog is an excellent model system for anterior cruciate ligament rupture in humans. Validation of the potentially novel loci identified here using our canine imputation panel would involve firstly confirming the genotypes and then functional studies, both of which are beyond the scope of this research study.

Imputation is a type of statistical inference, whereby genotype data are filled in based on observed haplotype patterns from WGS. As we have shown above, this is not 100% accurate, especially in dog breeds not well-represented in our WGS panel and, as a result, some significant associations identified in our imputation panel alone may be spurious. However, in general, we expect the effect of low imputation accuracy to be a reduction in power to detect significant associations, as the LD between an imputed marker and the causal variant will be reduced.

In summary, using our canine imputation panel of 24 million variants results in an increase in GWAS power, even for phenotypes that have multiple significant associations. The improvements to canine GWAS, especially for complex phenotypes, will not only further the field of canine genetics, but may also have beneficial implications for human medical genetics–especially for complex diseases, such as cancer and specific orthopedic diseases, for which the domestic dog is a good model organism [51].

## Materials and methods

### Whole genome sequences

The 365 whole genome sequences include 210 breed dogs (from 76 breeds), 107 village dogs (from 13 countries), and 28 wolves (S6 Table). 88 of these were sequenced at the Cornell University BRC Genomics Facility; others were sourced from public databases (S6 Table). Those sequenced at Cornell were run on an Illumina HiSeq2000 or Illumina HiSeq2500 and the reads were aligned to the CanFam3.1 reference genome using BWA version 0.7.8 [52]. Variants were called using GATK’s HaplotypeCaller [53–55]. Variant quality recalibration was done in GATK v3.5-0-g36282e4 using the semi-custom CanineHD variant sites [8] as a training set (known = false, training = true, truth = true, prior = 12.0). We included SNPs in the 99.9% tranche and removed sites with minor allele frequency (MAF) less than 0.5%. Phasing was done using Beagle 4.0 version r1399 [56], following the analysis pipeline used in the 1000 Genomes Project [57].

### Imputation panel

SHAPEIT v2.r790 [58] was used to phase the genotype data from 6,112 dogs as previously described [8] and then IMPUTE2 version 2.3.0 [59] was used to impute across these data. Imputation was only performed on the autosomes and chromosome X, not on the Y chromosome or mitochondrial SNPs. The final reference panel consists of 24.0 million variants, including 750,000 on the X chromosome, of which 20.33 million are SNPs and 3.67 million are indels.

### Imputation accuracy

276 purebred, 86 village, and 13 mixed-breed dogs were also genotyped on a second custom Illumina CanineHD 215k array, which contains 33,144 SNPs that are not on the 185k semi-custom CanineHD array but do feature in our imputed dataset. These 33,144 SNPs were used to determine the accuracy of our imputation across the 375 total dogs. Accuracy was calculated for each SNP as the number of sites that are correctly called in the imputed dataset divided by the total number of dogs. For example, if a G/C SNP was called G/G in 14 dogs, C/C in 10 dogs, and G/C in the remaining 351 dogs, then the imputation accuracy for that SNP is 93.6%. MAF was calculated for each SNP as the number of occurrences of the allele across all village, purebred, and mixed-breed dogs in the genotyped dataset. Imputation accuracy and MAF were plotted in Jupyter notebook [60] using Matplotlib library [61].

In addition, imputation accuracy was also calculated for a subset of these 375 dogs, namely 32 dogs of the four breeds (Yorkshire terrier, Maltese, German shepherd dog, Labrador retriever) most highly represented in the WGS reference panel (with 16, 13, 12, and 10 sequences, respectively). This was done to show the increase in imputation accuracy when using a reference panel containing dogs of the same breed as the genotyping dataset.

### Marker datasets

For the genotype data, individuals were run on a semi-custom Illumina CanineHD array of 185k SNPs, and quality control steps were performed as previously described [8]. For the imputed data, IMPUTE2 outputs were converted into PLINK [62] binary format, one for each chromosome.

### GWAS

We ran GWAS using a linear mixed model in the program GEMMA v 0.94 [63], with a MAF cut-off of 5% and using the Wald test to determine *P*-values. All LD plots were created using Matplotlib library [61] in Jupyter notebook [60].

For the imputed panel, GWAS was performed for each canine chromosome (CFA1-39) separately. The kinship matrix calculated using the array data in GEMMA was also used in the imputed GWAS for the same phenotype. The significance threshold was set to *P* = 1.0×10^−8^, based on thresholds calculated using the effective number of independent markers for human 1000 Genomes datasets [see 64] and given that LD in the domestic dog is more extensive than in humans [65].

### Body size

To identify QTLs associated with body size, we ran a GWAS of male breed-average weights (n = 1926) and heights (n = 1926), and individual sex-corrected weights (n = 3095), using both the semi-custom 185k CanineHD array data and the imputed panel data. Effect sizes were recorded from the GEMMA output, and MAFs and LD statistics were calculated using PLINK.

#### Male breed-average weight

The male breed-average weight data included 1926 dogs from 175 breeds with a maximum of 25 dogs per breed. The phenotype of male breed-average weight, in kg, was assigned to all dogs in the breed for the GWAS. We used weight to the power of 0.303 (weight^0.303^) to normalize the distribution of weights across the breeds, based on a Box-Cox transformation performed in R [66] using the package MASS [67].

#### Male breed-average height

For male breed-average height, we used the same data set as for weight above, that is, 1926 dogs from 175 breeds with a maximum of 25 dogs per breed. The phenotype of male breed-average height, in cm, was assigned to all dogs in the breed for the GWAS.

#### Individual sex-corrected weight

Individual sex-corrected weight GWAS was performed using 3,095 dogs including 417 village dogs and 427 mixed-breed dogs. The average raw weight in this individual dataset is 24.7kg, ranging from 1.6kg to 99.7kg. 164 breeds are represented, with 52 small breeds, 57 medium breeds, 34 large, and 21 giant breeds. Individual weights were sex-corrected by 16.47% (that is, female weights were increased by 16.47%), and also transformed, as above (weight^0.303^).

#### Covariate GWAS

GWAS of male breed-average weight using imputed variants shows the four most-associated loci are *HMGA2* (CFA10), *IGF1* (CFA15), *LCORL* (CFA3), and *SMAD2* (CFA7). In order to control for possible spurious associations, we ran an imputed GWAS, using male breed-average weights, including the four most-associated loci as covariates and then observed which of our significant associations remained.

#### Fine mapping

We used snpEff version 4.3T [68] to annotate our WGS variant file, using the pre-built CanFam3.1.86 database, and then used this to search for potential causal variants within specific LD regions.

#### Predicting body size

We used all the individual sex-corrected weights (n = 3,095) and specific body size QTLs as a training set, and then randomly set 20% of the weights to missing and used a Bayesian sparse linear mixed model (with a ridge regression/GBLUP fit) in GEMMA to predict these missing weights. We did this randomization and prediction 50 times, and then compared the actual weights to the predicted weights using a correlation coefficient.

#### Allelic heterogeneity

In order to reduce phenotypic noise, we again included all four most-associated QTLs as covariates in a follow-up GWAS of male breed-average weight and then, for those regions that still had residual association signal, we also included the most associated SNP (in addition to the four) as a covariate in the next GWAS. We continued this stepwise process of including the most-associated SNP in the next GWAS until there was no significant association signal in the respective region remaining.

### Blood phenotypes

Using previously published data of 39 phenotypes from complete blood count (CBC) and serum chemistry diagnostic panels of 353 dogs from a range of breeds [31], GWAS were performed using the imputed data in GEMMA, as described above. Results were compared to the published results using the semi-custom CanineHD array data [31].

### Cranial cruciate ligament disease

A quantitative GWAS was performed in GEMMA, as described above, using both the semi-custom CanineHD array data and the imputed data for the phenotype of cranial cruciate ligament disease (CCLD). The across-breed GWAS included 602 dogs total: 377 dogs had no rupture (controls), 141 dogs had a partial rupture, and 84 dogs had a complete rupture–this is the same dataset recently analyzed in a GWAS using FarmCPU [29]. Results from the imputed panel were directly compared to the array data.

## Supporting information

S1 Fig LD plots of the region around the male breed-average weight QTL intervals that have been fine-mapped. a) CFA3:41 near the gene *IGF1R*, b) CFA4:39 near the gene *STC2*, c) CFA4:67 near the gene *GHR*, d) CFA7:43 near the gene *SMAD2*, e) CFA10:8 near the gene *HMGA2*, f) CFA12:33, g) CFA15:41 near the gene *IGF1*, h) CFA18:20, i) CFAX near the gene *IGSF1*. Dashed lines show the significant interval (defined by *P*-values within two orders of magnitude of the most associated SNP). Asterisks show the location of the putative causal locus. Note that for d), f), g), and h) the putative causal locus is a deletion, retrogene insertion, SINE insertion, and retrogene insertion respectively, so these locations are labeled with asterisks at the top of the plot. Array genotypes are shown as o, imputed data are shown as +.
(TIF)

S2 Fig LD plots of the region around the male breed-average weight QTL intervals showing refinement of loci. a) CFA3:91 near the gene *LCORL* and *ANAPC13*, b) CFA7:30 near the gene *TBX19*. The significant interval, drawn with dashed vertical lines, is defined by *P*-values within two orders of magnitude of the most associated SNP. Array genotypes are shown as o, imputed data are shown as +. Black arrows point to the most significant SNP in the array GWAS and pink arrows point to the most significant variant in the imputed GWAS. Colors indicate amount of LD with the most significantly associated SNP, ranging from black (r^2^<0.2) to red (r^2^>0.8).
(TIF)

S1 Table Imputation accuracy calculated for each chromosome for village dogs, mixed-breed dogs, and purebred dogs. Average recombination rate (cM/Mb) calculated from Campbell et al. 2016 [69] for each chromosome is also shown.
(XLSX)

S2 Table *P*-values for body size GWAS QTLs. Values are shown for individual sex-corrected weight, male breed-average weight, and male breed-average height phenotypes with array genotypes and the imputed panel. Potentially novel body size loci are shown in bold.
(XLSX)

S3 Table Potentially novel body size loci at CFA9:12 and CFA26:7. a) Minor allele frequencies (MAF) by dog breed for the two potentially novel body size loci (CFA9:12,034,947 and CFA26:7,679,257) identified by imputed GWAS and b) proportion of dogs with different genotypes in different weight (male breed-average weight) classes for the two potentially novel body size loci (CFA9:12,034,947 and CFA26:7,679,257) identified by imputed GWAS.
(XLSX)

S4 Table Significant and nearly significant imputed GWAS results of blood phenotypes. Also shown is the location and *P*-value from the GWAS using the array genotype data.
(XLSX)

S5 Table Potentially novel CCLD locus at CFA20:42. a) Proportion of dogs of different phenotypes (control, partial rupture, complete rupture) with different genotypes at the CFA20:42,827,199 significant locus identified by imputed GWAS and b) frequencies, effect sizes and P-values for the CFA20:42,827,199 significant locus in different breeds.
(XLSX)

S6 Table List of Whole Genome Sequence (WGS) dog samples, breeds, and references. (XLSX)
