# Supplementary figures and images for: Imputation of canine genotype array data using 365 whole-genome sequences improves power of genome-wide association studies
Source: PLoS Genet. 2019 Sep 16;15(9):e1008003. doi: 10.1371/journal.pgen.1008003 (PMC6762211; doi:10.1371/journal.pgen.1008003)

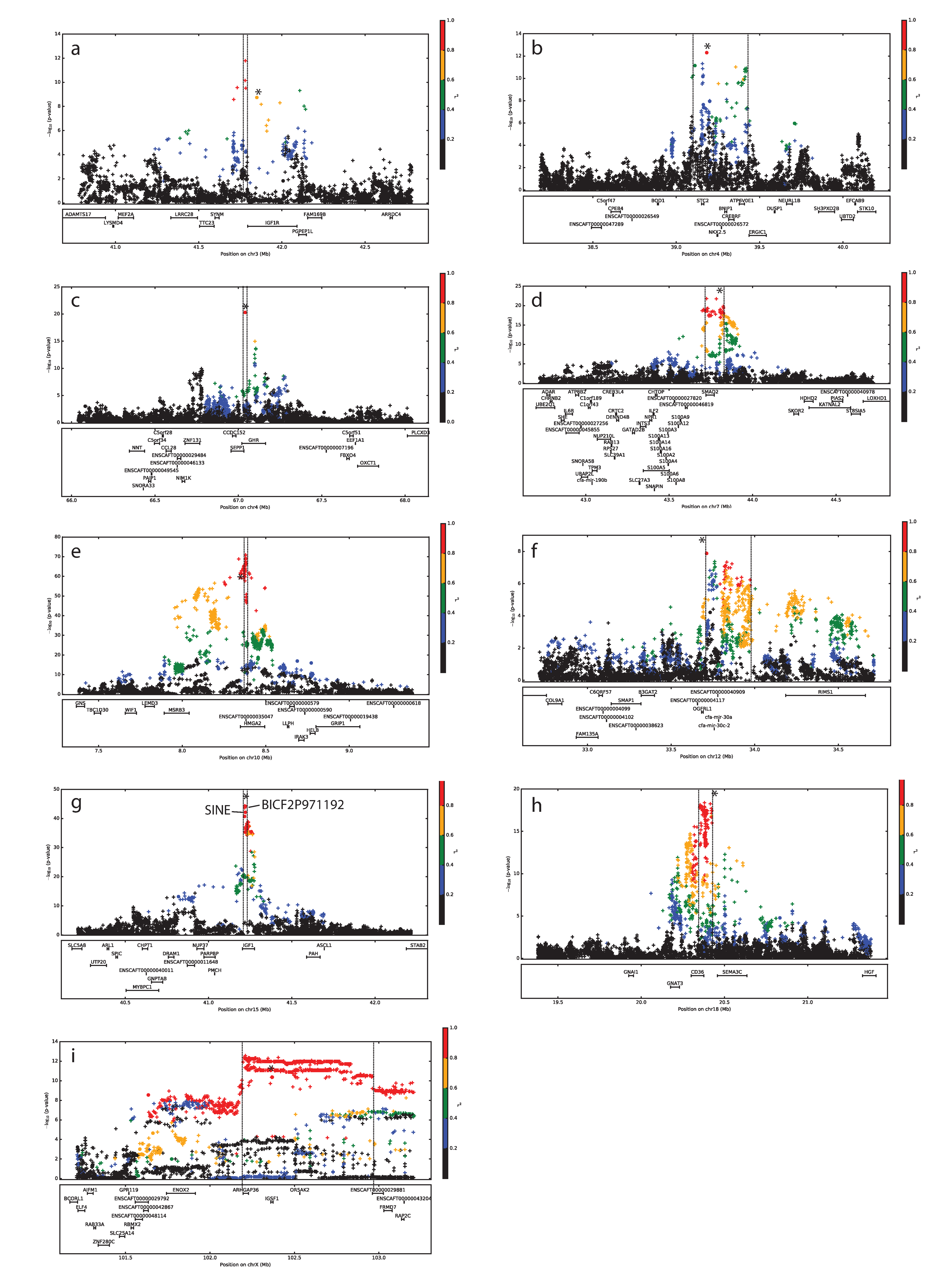

Supplement: S1 Fig — a) CFA3:41 near the gene IGF1R, b) CFA4:39 near the gene STC2, c) CFA4:67 near the gene GHR, d) CFA7:43 near the gene SMAD2, e) CFA10:8 near the gene HMGA2, f) CFA12:33, g) CFA15:41 near the gene IGF1, h) CFA18:20, i) CFAX near the gene IGSF1. Dashed lines show the significant interval (defined by P-values within two orders of magnitude of the most associated SNP). Asterisks show the location of the putative causal locus. Note that for d), f), g), and h) the putative causal locus is a deletion, retrogene insertion, SINE insertion, and retrogene insertion respectively, so these locations are labeled with asterisks at the top of the plot. Array genotypes are shown as o, imputed data are shown as +. (TIF) [file pgen.1008003.s001.tif]

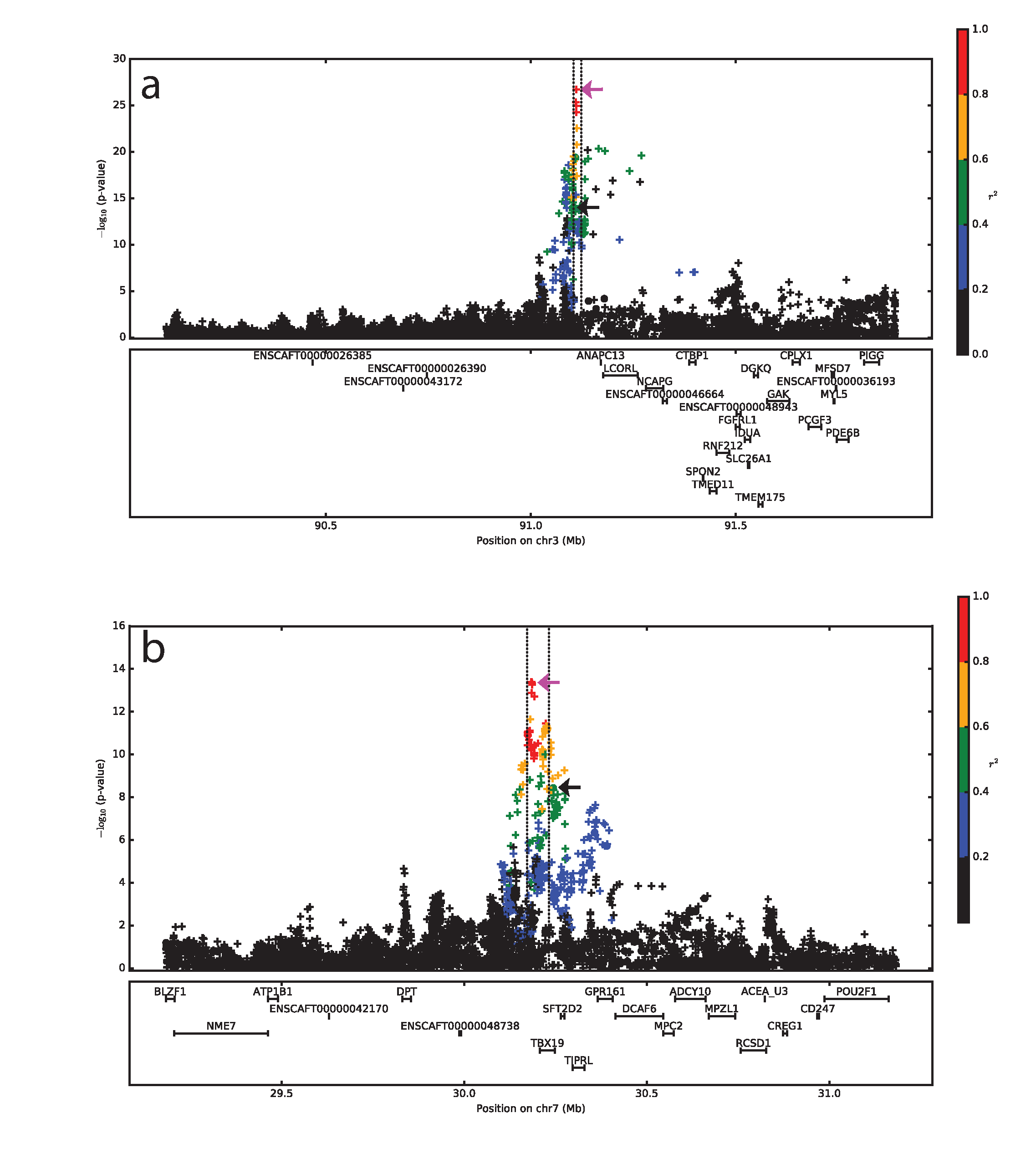

Supplement: S2 Fig — a) CFA3:91 near the gene LCORL and ANAPC13, b) CFA7:30 near the gene TBX19. The significant interval, drawn with dashed vertical lines, is defined by P-values within two orders of magnitude of the most associated SNP. Array genotypes are shown as o, imputed data are shown as +. Black arrows point to the most significant SNP in the array GWAS and pink arrows point to the most significant variant in the imputed GWAS. Colors indicate amount of LD with the most significantly associated SNP, ranging from black (r2<0.2) to red (r2>0.8). (TIF) [file pgen.1008003.s002.tif]
